# Magnitude and Complexity of Rectal Mucosa HIV-1-Specific CD8+ T-Cell Responses during Chronic Infection Reflect Clinical Status

**DOI:** 10.1371/journal.pone.0003577

**Published:** 2008-10-30

**Authors:** J. William Critchfield, Delandy H. Young, Timothy L. Hayes, Jerome V. Braun, Juan C. Garcia, Richard B. Pollard, Barbara L. Shacklett

**Affiliations:** 1 Department of Medical Microbiology and Immunology, University of California Davis, Davis, California, United States of Amerca; 2 Department of Statistics, University of California Davis, Davis, California, United States of Amerca; 3 Department of Medicine, Division of Gastroenterology, School of Medicine, University of California Davis, Sacramento, California, United States of America; 4 Department of Medicine, Division of Infectious Diseases, School of Medicine, University of California Davis, Sacramento, California, United States of America; University of California San Francisco, United States of America

## Abstract

**Background:**

The intestinal mucosa displays robust virus replication and pronounced CD4+ T-cell loss during acute human immunodeficiency virus type 1 (HIV-1) infection. The ability of HIV-specific CD8+ T-cells to modulate disease course has prompted intensive study, yet the significance of virus-specific CD8+ T-cells in mucosal sites remains unclear.

**Methods and Findings:**

We evaluated five distinct effector functions of HIVgag-specific CD8+ T-cells in rectal mucosa and blood, individually and in combination, in relationship to clinical status and antiretroviral therapy (ART). In subjects not on ART, the percentage of rectal Gag-specific CD8+ T-cells capable of 3, 4 or 5 simultaneous effector functions was significantly related to blood CD4 count and inversely related to plasma viral load (PVL) (p<0.05). Polyfunctional rectal CD8+ T-cells expressed higher levels of MIP-1β and CD107a on a per cell basis than mono- or bifunctional cells. The production of TNFα, IFN-γ, and CD107a by Gag-specific rectal CD8+ T-cells each correlated inversely (p<0.05) with PVL, and MIP-1β expression revealed a similar trend. CD107a and IFN-γ production were positively related to blood CD4 count (p<0.05), with MIP-1β showing a similar trend. IL-2 production by rectal CD8+ T-cells was highly variable and generally low, and showed no relationship to viral load or blood CD4 count.

**Conclusions:**

The polyfunctionality of rectal Gag-specific CD8+ T-cells appears to be related to blood CD4 count and inversely related to PVL. The extent to which these associations reflect causality remains to be determined; nevertheless, our data suggest a potentially important role for mucosal T-cells in limiting virus replication during chronic infection.

## Introduction

A significant body of work has demonstrated the contribution of HIV-specific CD8+ T-cell responses to immunological control of HIV infection [1]–[3]. However, the relationship between cytotoxic T-lymphocyte (CTL) response magnitude and clinical parameters has proven controversial. Mathematical models based upon the ability of CTL to clear virally infected cells predicted an inverse relationship between blood CTL frequency and HIV plasma viral load in the absence of antiretroviral therapy [4]. Some reports, using MHC class I tetramers and/or Elispot assays to quantify HIV-specific T-cell responses, did in fact reveal such an inverse relationship [5], [6]. However, subsequent studies did not produce a consensus on this point: one study reported a positive correlation between plasma viral load and the total HIV-specific, Env-, and Nef-specific CD8+ T-cell frequency [7], while at least two others found no numerical relationship [8], [9]. Recently, additional light has been shed on this issue by reports documenting a unique relationship between the breadth and/or magnitude of Gag-specific CD8+ T-cell responses (but not Env, Pol or Nef-specific responses) and plasma viremia and/or blood CD4 count [10]–[16]. Mechanisms proposed to explain the immunodominance of Gag-specific responses include early recognition of infected cells by Gag-specific CTL [17] and immune pressures exerted by CTL specific for conserved Gag functional domains [18].

Given the ambiguous relationship between HIV-specific T-cell response magnitude and clinical parameters, investigators have sought new combinations of immunological markers whose expression might more reproducibly correlate with clinical status. The advent of multiparameter flow cytometry, allowing the simultaneous measurement of multiple effector molecules, has led to an emphasis on T-cell response “quality” or “functional heterogeneity” rather than “quantity” or “magnitude” of IFNγ responses alone [19]. While it could be argued that the measurement of five functions will not necessarily provide more relevant information than the measurement of a single function, recent findings have in fact demonstrated the added value of this approach. HIV long-term nonprogressor status was associated with the ability of HIV-specific CD4+ T-cells to produce both IL-2 and IFNγ [20]. Conversely, in individuals with progressive disease, HIV-specific CD4+ T-cell responses were skewed towards production of IFNγ in the absence of IL-2 [21]. In one study, the ability to mount 4 and 5 functions simultaneously (i.e., IFNγ, TNFα, IL2, MIP-1β and CD107a) was a characteristic of HIV-specific CD8+ T-cells in long-term nonprogressors [22]. In a related study, the control of HIV-1 replication in HLA-B27 subjects was associated with the ability of B27-KK10-specific CD8+ T-cells to mount multiple effector functions [23]. T-cell responses associated with successful vaccines also support the clinical relevance of polyfunctionality. Humans immunized with vaccinia virus generate polyfunctional virus-specific CD8+ T-cells [24], and vaccine-induced protection of mice challenged with *Leishmania major* is accompanied by T-helper type 1 cells that are able to produce multiple cytokines [25].

Most prior studies of HIV-specific cell-mediated immunity have assessed responses only in the peripheral blood compartment; little information is available concerning HIV-specific T-cell responses in mucosal tissues. In a previous study focused primarily on IFN-γ production, we reported that rectal CD8+ T-cell responses to Gag were immunodominant (as compared to Pol and Env) in chronically infected subjects not on antiretroviral therapy with a broad range of viral loads [26]. We also found that the production of IFN-γ by rectal HIV-gag CD8+ T-cells was positively associated with blood CD4 count during chronic infection [26], and began to explore the ability of rectal CD8+ T-cells to mount multiple effector responses, including CD107a, TNF-α and IFN-γ. In the present study, we have extended these analyses to include five effector functions, both individually and combination, building upon our existing cohort of individuals with chronic HIV infection.

In the present work, we have analyzed paired blood and rectal biopsies from chronically infected patients for five distinct responses to HIVgag stimulation: production of the cytokines IFN-γ, IL-2, TNF-α, the chemokine MIP-1β, and the cytolytic granule constituent CD107a, which is an indirect marker for cytotoxic potential [27]. We present a detailed analysis of the relationship between each individual effector function and clinical parameters, as well as the relationship between T-cell polyfunctionality and clinical parameters. The patient group includes 15 individuals not on antiretroviral therapy (ART) with viral loads ranging from <50 to >50,000 copies/ml, as well as 13 patients on ART for at least one year. We also introduce a new statistics-based approach to determining when an antigen-specific response is significantly different from background; the aim of this tool is to remove potential bias and facilitate data interpretation in instances when cell number is variable and background high, as is frequently the case with mucosal samples.

Using an expanded panel of response parameters coupled with a new and rigorous statistical approach to evaluating when a response is significant (see Materials & Methods), here we report on multiple significant associations between CD8+ T-cell responses in rectal mucosa and both plasma viral load and blood CD4 count. Such associations were not evident when evaluating peripheral blood CD8 responses in relationship to plasma viral load or blood CD4 count in the same patients. We also report that HIV-specific CD8+ T-cell responses in rectal mucosa of patients on long-term suppressive ART are characterized by low magnitude and a marked qualitative shift towards monofunctional responses when compared to patients not on ART. Thus, rectal mucosal CD8+ T-cells are not only capable of potent and diversified effector responses, but these responses bear a relationship to clinical status during chronic HIV infection.

## Results

### Characteristics of Study Subjects

Plasma viral load, blood CD4 count, and other clinical information for the 36 study participants are shown in Table 1. Study participants included 15 seropositive individuals not on ART, 13 seropositive subjects on ART, and 8 seronegative volunteers. Several of these patients participated in an earlier study of mucosal T-cell responses [26]; however, fresh biopsy samples were obtained for the present work. For seropositive subjects, the number of years since initial HIV-1 seropositivity ranged from 1 to 25 years. Time since seroconversion averaged 8.3 years for patients not on ART and 10.7 years for patient on ART, a difference which was not statistically significant (Mann Whitney test, 2-tailed, *p*>0.05). The subject pool was primarily Caucasian (56%) and African American (33%), and the gender distribution was approximately two-thirds male and one-third female. Among seropositive subjects not on ART, viral loads ranged from <50 to 89,700 copies per ml plasma, and blood CD4 counts ranged from 304 to 883 cells per µl blood (Table 1). Among the 13 subjects on ART, the time since ART initiation ranged from 1–15 years; drug combinations at the time of biopsy are given in Table 1. Three of the 13 subjects on ART did not have fully suppressed plasma viremia. Seronegative subjects were confirmed to be uninfected by HIV-1 antibody testing and plasma viral load testing.

**Table 1:** Patient Characteristics.

Patient ID^a^	Gender^b^	Ethni-city^c^	HLA Class I Genotype	Blood CD4 (cells/mm^3^)	Plasma VL (RNA copies/ml)	Yrs HIV^+^	ART (at time of biopsy)^d^	ART Duration (minimum time)
6	M	AA	A2/A74/B49/B81	367	1,130	17	No	NA
32	F	H	A3/A11/B7/B27	740	5,370	11	No	NA
50	F	AA	A30/B8/B27	619	281	18	No	NA
56	M	C	A1/A29/B8/B44	304	76,000	3	No	NA
58	M	C	A1/A2/B51/B58	538	89,700	7	No	NA
60	M	C	A2/A3/B14/B39	669	703	25	No	NA
62	F	C	A1/A2/B37/B57	571	12,700	5	No	NA
65	M	C	A1/A2/B7/B8	719	63,800	1	No	NA
66	M	C	A23/A68/B8/B51	481	83,900	1	No	NA
67	M	C	A11/A24/B13/B52	715	27,100	1	No	NA
71	M	H	A11/A31/B15/B51	463	34,900	9	No	NA
78	M	H	A2/A68/B18/B57	636	109	1	No	NA
79	F	C	A2/A30/B14/B52	553	7,170	11	No	NA
85	M	C	A1/A68/B27/B57	883	<50	10	No	NA
98	F	C	A2/A25/B8/B18	608	69,600	4	No	NA
51	F	AA	A30/A68/B45	358	11,000	12	FTC+TDF+EFV	10 yr
54	T	C	A24/A68/B51	512	<50	17	EFV+3TC+TDF	1 yr
57	M	C	A1/B7/B8	442	<50	7	AZT+3TC+EFV	4 yr
63	F	C	A1/A2/B8/B44	464	<50	4	TDF+EFV+ABC+AZT	4 yr
64	M	C	A11/A24/B7/B44	851	<50	24	ABC+3TC+ATV+RTV	15 yr
77	M	AA	A2/A33/B53/B57	408	<50	17	FTC+TDF+RTV+ATV	3 yr
80	M	C	A2/A31/B44/B51	927	<50	17	FTC+TDF+RTV+ATV	3 yr
81	M	AA	A68/B7/B58	527	557	6	DDI+TDF+RTV+FPV	4 yr
83	M	AA	A23/A74/B15/B18	287	<50	1	DDI+TDF+LPV+RTV	2 yr
88	M	C	A2/A29/B14/B49	316	<50	6	AZT+3TC+EFV	4 yr
89	I	AA	A3/B7/B49	434	7,660	6	ABC+3TC+ATV	2 yr
92	M	C	A2/A26/B40/B44	545	<50	19	FTC+TDF+LPV+RTV	6 yr
93	F	C	A1/B8/B39	809	<50	3	3TC+TDF+RTV+ATV	3 yr
27	M	AA	A23/A24/B15/B27	837	<50	NA	HIV neg	NA
74	M	AA	A30/A68/B15/B57	322	<50	NA	HIV neg	NA
86	F	AA	A23/B18/B49	670	<50	NA	HIV neg	NA
87	F	C	A2/A24/B7/B57	742	<50	NA	HIV neg	NA
91	F	C	A2/B8/B35	714	<50	NA	HIV neg	NA
94	F	As	A11/A33/B44/51	228	<50	NA	HIV neg	NA
96	M	AA	ND	1396	<50	NA	HIV neg	NA
100	M	AA	A1/A30/B44/B49	609	<50	NA	HIV neg	NA

a ID, identification number.

b M, male; F, female; T, transgender; I, intersex.

c AA, African-American; As, Asian; C, Caucasian; H, Hispanic.

d ABC, Abacavir; ATV, Atazanavir; AZT, Azidothymidine; DDI, Didanosine; EFZ, Efavirenz; FPV, Fosamprenavir; FTC, Emtricitabine; LPV, Lopinavir; RTV, Ritonavir; TDF, Tenofovir Disoproxil Fumarate; 3TC, Lamivudine.

### Flow cytometry gating

**Figure 1:**
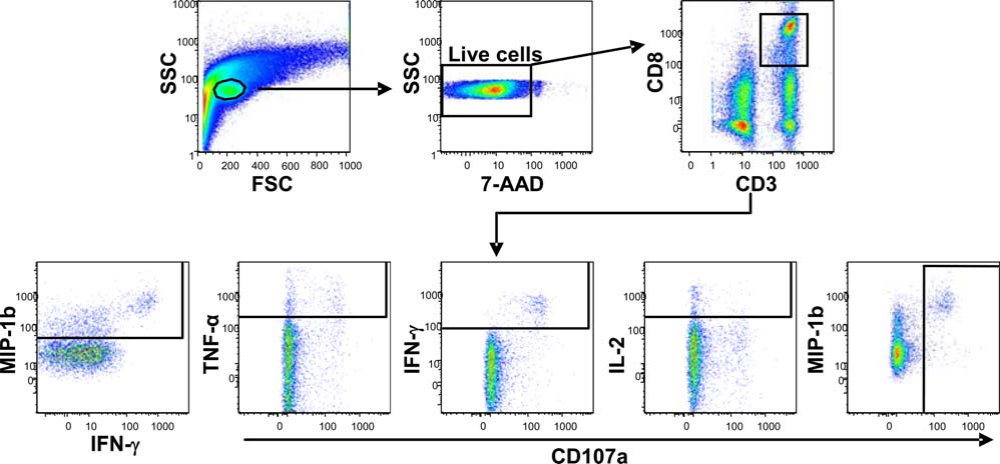
Flow cytometry gating method used in the analysis of CD8+ T-cell responses. Shown is a typical rectal mucosal cell preparation that was stimulated with HIVgag peptides. After initial estimation of a lymphocyte gate based on forward versus side scatter, viable cells (7-AAD negative) were carried forward for selection of CD3+CD8+ cells. The resulting CD3+CD8+ population was further gated based on positivity for each of the 5 functional responses measured: MIP-1β, TNF-α, IFN-γ, IL-2 and CD107a. The frequencies of cells in these populations were corrected using background values (costimulation only) and then compiled and analyzed directly. For more detailed analyses, the 5 populations were instead entered into Boolean gating analysis to generate 31 populations consisting of all possible combinations of functionality (the 32^nd^ population representing cells negative for all 5 functions was ignored). Background correction was applied after Boolean gating (see Methods).

The gating strategy used to analyze data from the ICS assay of a typical rectal MNC sample stimulated with HIVgag peptides plus costimulatory antibodies is illustrated in Figure 1. A lymphocyte gate based on forward/side scatter was followed by a viability gate (7-AAD negative) and selection of cells positive for both CD3 and CD8. These cells were further subdivided into individual populations consisting of cells positive for MIP-1β, IFN-γ TNF-α, IL-2, and CD107a. Values from these populations were either analyzed directly or the populations further subdivided into 31 possible combinations of functions [22], [28]. In either case, values were background-corrected as described in Materials and Methods, using values from cells treated with costimulatory antibodies alone. For phenotyping of rectal and peripheral CD8+ T-cells, unstimulated cells underwent the same gating strategy except that the CD3+CD8+ population was further divided based upon expression of phenotypic markers rather than response markers.

### Rectal CD4+ T-cell percentages reflect peripheral blood CD4 percentages in the group on ART

**Figure 2:**
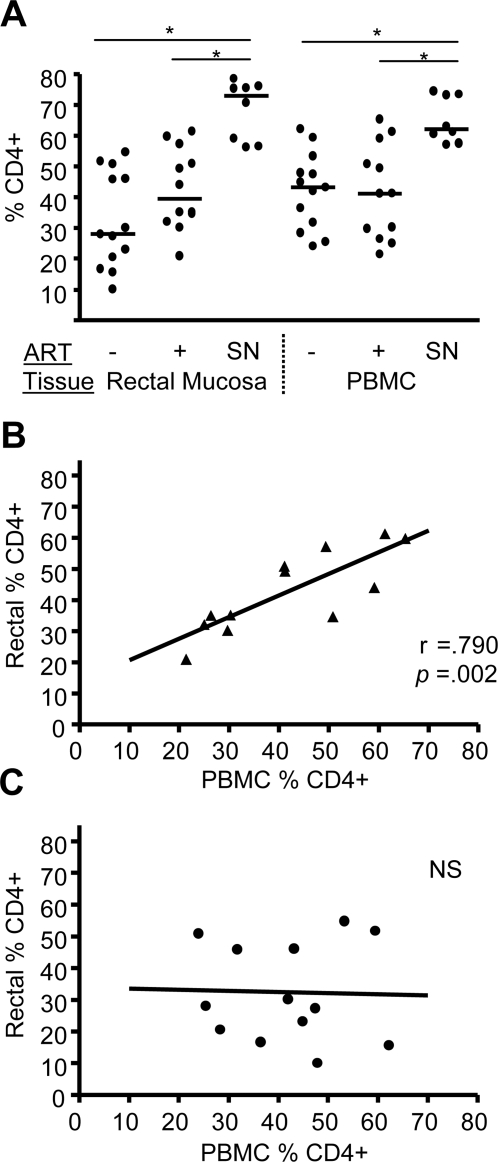
CD4+ T-cell percentages in rectal mucosa and peripheral blood. (A) CD4 values for rectal mucosa and peripheral blood are indicated as a percentage of CD3+ cells for subjects on and off ART, and seronegative subjects. The horizontal line denotes the median. Statistically significant differences (p<0.05) within each tissue are denoted by a line with asterisk. SN, seronegative. (B & C) Correlation analysis (Spearman) of CD4+ T-cell percentages from each tissue for patients on ART (Part B, filled triangles) and not on ART (Part C, filled circles). Lines represent best fits as estimated by linear regression.

The dynamics of CD4+ T-cell restoration in mucosal tissues following initiation of ART is an area of current interest [29], [30]. We therefore measured CD4+ T-cells as a percentage of CD3+ T-cells and explored the relationship between CD4+ T-cell frequencies in rectal mucosa and blood in a cross-sectional analysis of subjects either on or off ART, as shown in Fig. 2. CD4+ T-cell levels in both rectal mucosa and PBMC were lower in HIV-infected individuals, irrespective of ART status, compared to seronegative controls (Fig 2A). Although rectal CD4+ T-cell percentages were frequently lower than blood CD4+ T-cell percentages in patients not on ART, a comparison of CD4+ T-cell percentages between rectal mucosa and PBMC in both patient groups, using paired T-tests, did not reveal significant differences between tissues. Of note, the group on ART had a higher median rectal CD4+ T-cell frequency (39.6%) compared to the group not on ART (28.0%), though this difference was not statistically significant. Moreover, the group on ART displayed a significant positive correlation between rectal and peripheral CD4 percentages (Fig. 2B, p = 0.001), while the group not on ART failed to show such a relationship (Fig. 2C). The group on ART also displayed an inverse correlation (*p*<0.05) between rectal CD4 percentage and plasma viral load (data not shown).

### Rectal CD8+ responses are robust compared to PBMC, but are strongly attenuated by ART

**Figure 3:**
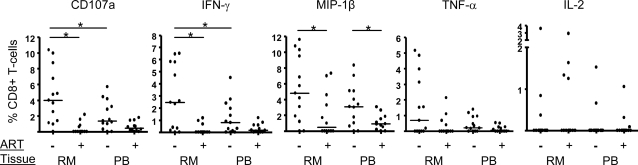
Individual responses to HIVgag peptides mounted by rectal mucosal and peripheral CD8+ T-cells. Following peptide stimulation, cells were evaluated for five distinct functions (i.e., production of IFN-γ, TNF-α, IL-2, MIP-1β and CD107a) using flow cytometry. Values shown are the percentage of CD8+ T-cells positive for a given function, with medians indicated by a horizontal line. Statistically significant differences between groups (*p*<0.05) are denoted by a line with an asterisk. RM, rectal mucosa, PB, peripheral blood, ‘−’, not on ART; ‘+’, on ART.

All Gag-specific responses reported in this study were first evaluated for significance, in relation to responses associated with exposure to costimulatory antibodies alone, using a statistical test rather than a simple cutoff value (see Materials & Methods). Responses to HIVgag peptide stimulation (i.e., percentages of responding CD8+ T-cells) were first analyzed individually, as summarized in Figure 3. For CD107a, IFN-γ, MIP-1β, and TNF-α, responses were vigorous in both tissue compartments in patients not on ART. IL-2 production by CD8+ T-cells was weak or absent in most subjects and in both tissues. In the subject group not on ART, rectal responses were particularly robust compared to blood; for CD107a and IFN-γ the differences between median response magnitudes when comparing rectal mucosa to blood were greater than 2-fold and were statistically significant. As predicted, mucosal T-cell responses in patients on ART were lower in magnitude than in the untreated group. Furthermore, the difference in response magnitude in rectal mucosa between patients off and on ART was statistically significant for three functions: CD107a, IFN-γ, and MIP-1β (Figure 3). Although response magnitudes in PBMC showed a similar trend, only one function, MIP-1β production, showed a significant difference when comparing the two treatment groups. Thus, ART was associated with a particularly striking decrease in the magnitude of HIV-specific T-cell effector functions in rectal mucosa.

### Rectal but not peripheral CD8+ T-cell response magnitudes in subjects not on ART correlate with plasma viral load and blood CD4 count

**Figure 4:**
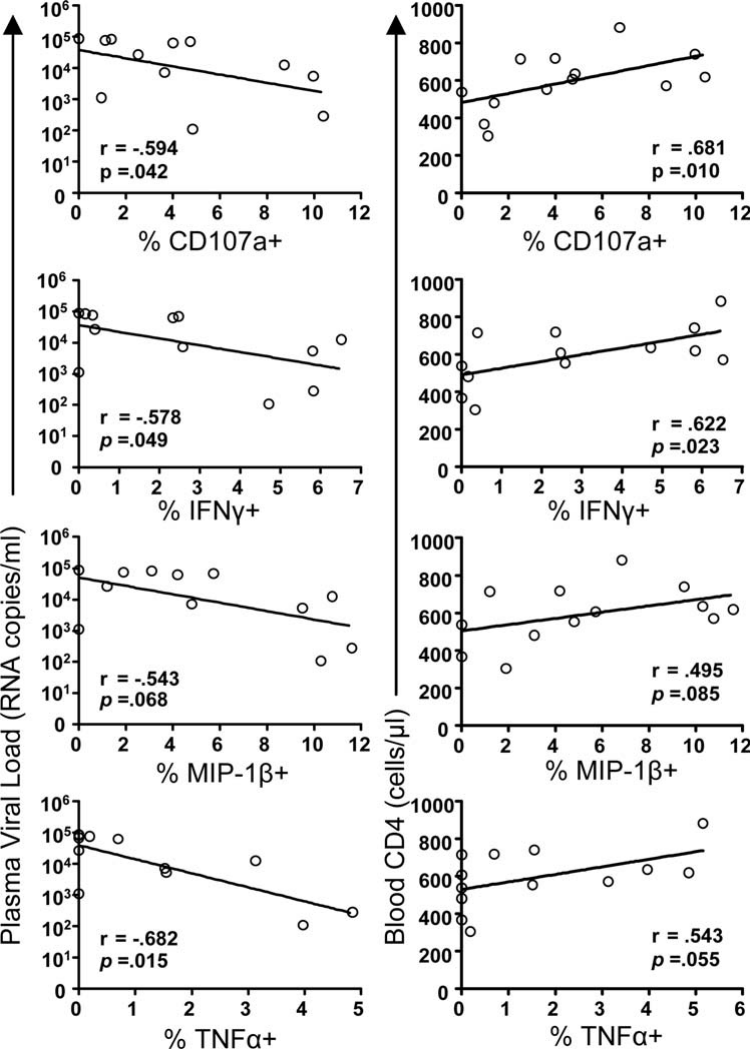
Correlation (Spearman) of rectal CD8+ T-cell responses to plasma viral load or blood CD4 count in patients not on ART. CD8+ T-cell responses (shown as percentages of cells producing cytokines/chemokines or CD107a, as in Fig. 3), were plotted against viral load and CD4 percentages. Results for Spearman r value and level of significance are shown. Lines represent best fits as estimated by linear regression. Comparable relationships between PBMC functional responses and clinical parameters are not shown, as none were statistically significant.

As shown in Fig. 3, the group of subjects not on ART displayed a broad range of response magnitudes in both rectal mucosa and blood for each individual function, with the exception of IL-2. For example, net rectal CD107a response values ranged from zero to 10.4%. To investigate whether response magnitudes were related to virus replication or clinical status, we looked for correlations with plasma viral load and blood CD4 count (Fig. 4). Strikingly, in rectal mucosa, three of the five Gag-specific CD8+ T-cell responses measured (i.e., CD107a, IFN-γ, and TNF-α) were found to be inversely correlated with plasma viral load (*p*<0.05). Furthermore, CD107a and IFN-γ responses in rectal mucosa were positively correlated with blood CD4 count (*p*<0.05). In contrast, Gag-specific response magnitudes in PBMC were not significantly related to viral load or to blood CD4, and rectal CD8+ T-cell responses did not show a significant relationship to rectal CD4 percentage (data not shown). These findings suggest that, in individuals with chronic infection in the absence of ART, HIV-specific T-cell responses measured in rectal mucosa may be a stronger correlate of viral replication and immune status than the corresponding responses measured in peripheral blood.

### CD8+ T-cells in rectal mucosa display complex functionality which is diminished with ART

**Figure 5:**
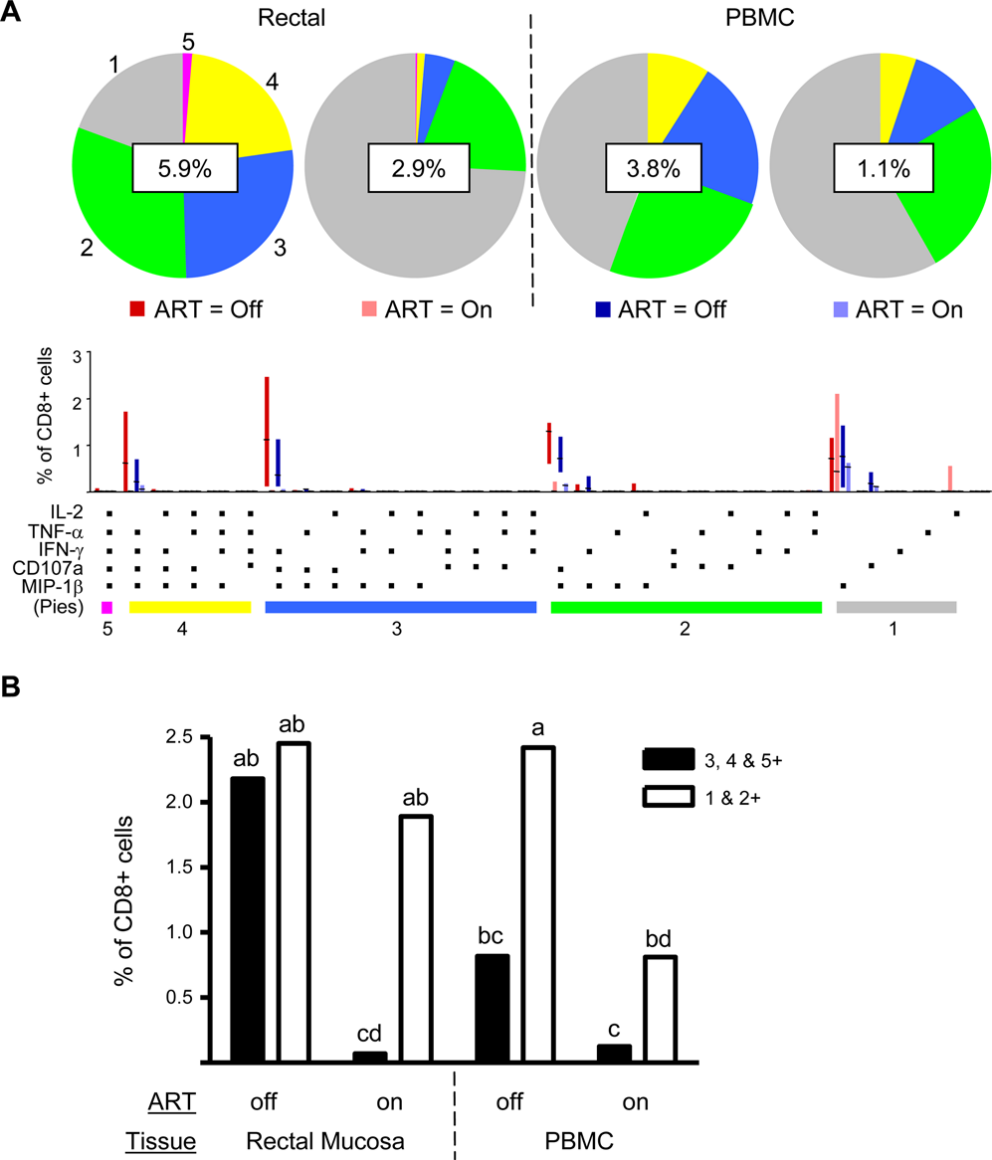
Detailed HIVgag response profile for CD8+ T-cells in rectal mucosa and blood. (A) Response profiles were generated using Boolean analysis of 5 functional response gates, resulting in 31 separate categories as described in the text. The percentage value shown in the center of each pie diagram denotes the total magnitude of CD8+ T-cells responding in any way (i.e., a summation across all 31 categories). Individual color-coded pie slices represent the fraction of cells within the total responding population displaying 5, 4, 3, 2, or 1 function(s), beginning at ‘12 o-clock’ and moving clock-wise, as compiled from the bar graph data. The bar graph provides fine detail on frequencies within each of the individual 31 categories; interquartile ranges and medians are shown. (B) Results from linear mixed model analysis of HIVgag-specific responses. ART status, tissue type, and polyfunctionality were treated as fixed effects (see Methods). Polyfunctional cells (open bars) were defined as cells positive for 3, 4, or 5 functions, and non-polyfunctional cells (closed bars) were defined as cells positive for 1 or 2 functions. Letters are statistical notations; bars that do not share a common letter are significantly different (p<0.05).

In the subject group not on ART, the enhanced HIVgag responsiveness of rectal CD8+ T-cells in comparison to PBMC, and the significant relationship of rectal responses with plasma viral load and blood CD4 count (Fig. 4), led us to ask whether the ability of rectal CD8+ T-cells to mount complex polyfunctional responses might also be related to clinical parameters. To analyze response complexity, we subjected the antigen-specific response data (derived as shown in Fig. 1) to Boolean gating analysis, generating 31 separate response categories consisting of all possible combinations of functions. The results from this analysis, generated using SPICE software, are illustrated in Fig. 5. In agreement with previous reports [22], this analysis resulted in the distribution of cells into a small number of distinct response groups. The most complex group, which was observed at low frequency, was that displaying all 5 functions. Other major groups, shown from left to right in Fig. 5B, were the 4+ group (lacking IL-2), the predominant 3+ group (lacking IL-2 and TNF-α), the predominant 2+ group (expressing only CD107a and MIP-1β), and the predominant 1+ group expressing only MIP-1β.

The mean total percentage of rectal CD8+ T-cells responding to HIVgag stimulation in any way (i.e., all 31 non-overlapping categories combined) was 5.9% for the group not on ART. The pie charts and bar graphs provide finer details illustrating that nearly one-half of the rectal response in patients not on ART was comprised of polyfunctional cells capable of 3, 4, or 5 responses simultaneously. In contrast, in the group on ART, the mean percentage of rectal CD8+ T-cells responding to HIVgag stimulation was 2.9%. Even more strikingly, cells producing 3, 4, or 5 responses accounted for less than 10% of the responding population, as shown in the pie chart. Thus, in this cross-sectional comparison of patients on and off ART, ART was associated with a striking absence of polyfunctionality in rectal mucosa.

In PBMC of patients not on ART, the mean percentage of HIVgag-specific CD8+ T-cells was lower than in rectal mucosa (3.8% vs. 5.9%), and approximately 30% of this response was derived from cells capable of at least 3 responses. In the ART group, the mean percentage of responding cells was 1.1%, fewer than 20% of which were polyfunctional. Figure 5B is a graphical representation of the results of linear mixed model analysis for the data shown in Figure 5A. Data for each tissue and patient group are summarized by individual bars, and letter codes are used to identify statistically significant differences between groups. Polyfunctional cells, capable of 3, 4 or 5 functions, are represented by open bars, and cells capable of only 1 or 2 functions are represented by filled bars. As shown in the figure, HIVgag-specific responses in patients on ART showed reduced magnitude and complexity as compared to responses in patients not on ART. Notably, CD8+ T-cell response complexity was far more sensitive to ART in rectal mucosa than in PBMC in this cross-sectional data set.

### Response complexity is related to blood CD4 count and plasma viral load

The utility of Boolean gating analysis lies not only in the ability to assemble a detailed view of responding CD8+ T-cell populations (Fig. 5), but also in the opportunity to look within selected response categories for relationships between responses and clinical status. We therefore asked whether any single response category in blood or rectal mucosa, or combination of categories, was significantly related to blood CD4 count or plasma VL. These analyses were limited to those T-cell response categories for which at least 50% of the responses were greater than zero, and the analysis of subjects was limited to those not on ART, since those on ART had a high percentage of zero values.

**Figure 6:**
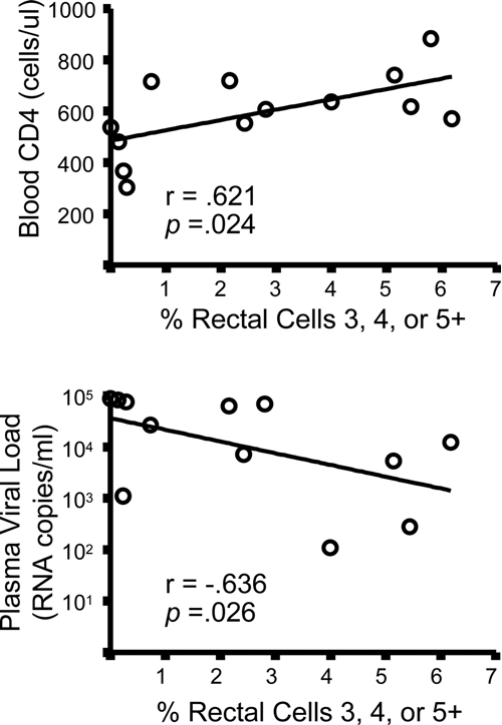
Correlation (Spearman) of polyfunctional rectal CD8+ T-cell responses and plasma viral load or blood CD4 count. Polyfunctionality was defined as the summed frequencies of the 5+ and predominant 4+ & 3+ groups (as shown in the bar graph, Fig. 4). This derived value was plotted (X-axis) against blood CD4 count or plasma viral load (Y-axis). Results for r and level of significance are shown. Comparable relationships between PBMC polyfunctional responses and clinical parameters are not shown, as they were not statistically significant.

Rectal, but not peripheral, Gag-specific CD8+ T-cell responses within certain functional categories showed significant correlations with clinical status. The strongest of these relationships was observed when the percent response was summed across the three predominant polyfunctional categories and the summed values were plotted against blood CD4 count or plasma viral load. Figure 6 shows results of this analysis; the total of 3+, 4+, and 5+ CD8+ T-cell frequencies was positively correlated with blood CD4 count (r = 0.621, *p* = .024) and negatively correlated with plasma viral load (r = −0.636, *p* = .026). In contrast, the summed 1- and 2-function responses showed no relationship to blood CD4 or plasma viral load (not shown). Additional significant correlations (*p*<.05) included mucosal 4 and 3-function CD8 responses in relation to blood CD4 count; mucosal 4-function CD8 responses in relation to plasma viral load; and the summed 1+ through 5+ mucosal Gag-specific CD8 response in relation to blood CD4 count and plasma viral load (data not shown). Although similar statistical analyses were performed for blood CD8+ T-cell responses, we did not identify any significant relationships between total blood CD8 responses or any subset of blood CD8 responses and either plasma VL or blood CD4 count (not shown). In addition, functionality of rectal CD8+ T-cell responses failed to exhibit any significant correlation with rectal CD4 frequency as a percent of CD3+ T-cells (not shown).

### The production of MIP-1β and CD107a on a per-cell basis intensifies as cell functionality increases

**Figure 7:**
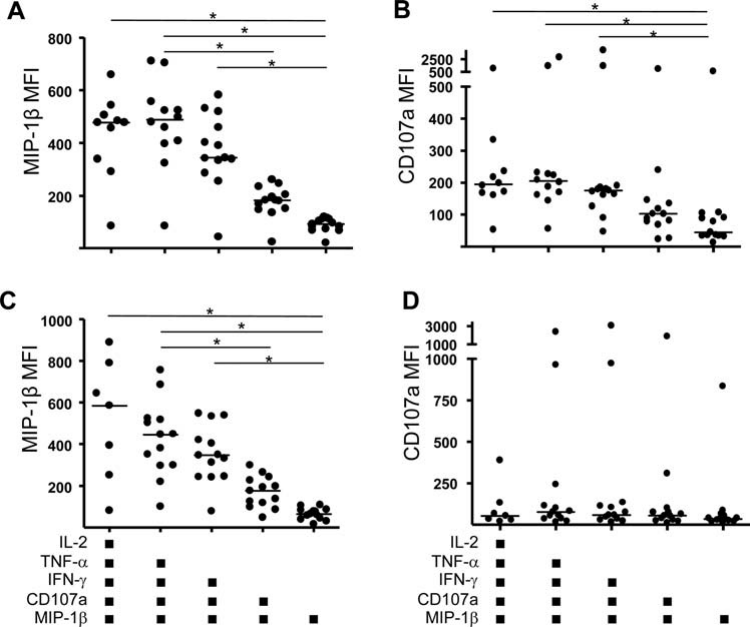
Median fluorescence intensity (MFI) of MIP-1β and CD107a staining of HIVgag specific CD8+ T-cells in relation to response category. (A & B) Rectal mucosa. (C & D) Peripheral blood. Statistically significant differences (p<0.05) are denoted by a line with an asterisk.

Polyfunctional CD8+ T-cells have previously been demonstrated to produce more IFN-γ on a per-cell basis than cells of lower functionality [23]–[25]. In the present data set, the expression of MIP-1β and CD107a were highly conserved across the predominant response categories for subjects not on ART (Fig. 5). This pattern of conserved expression afforded the opportunity to evaluate the median fluorescence intensity (MFI) of these factors across the spectrum from single-positive to 5-function positive cells in both PBMC and rectal mucosa. MFI values were adjusted by subtracting the MFI of cells negative for expression. As shown in Figure 7, MIP-1β and CD107a MFIs in rectal CD8+ T-cells (Figs. 7A & 7B) were significantly increased at higher levels of response complexity compared to single positive cells. In peripheral blood (Figs. 7C & 7D), MIP-1β showed the same pattern as in rectal mucosa, while CD107a MFI showed no changes across the response categories.

### The expression of activation markers and perforin and granzyme on CD8+ T-cells reveals tissue and ART-dependent effects

**Figure 8:**
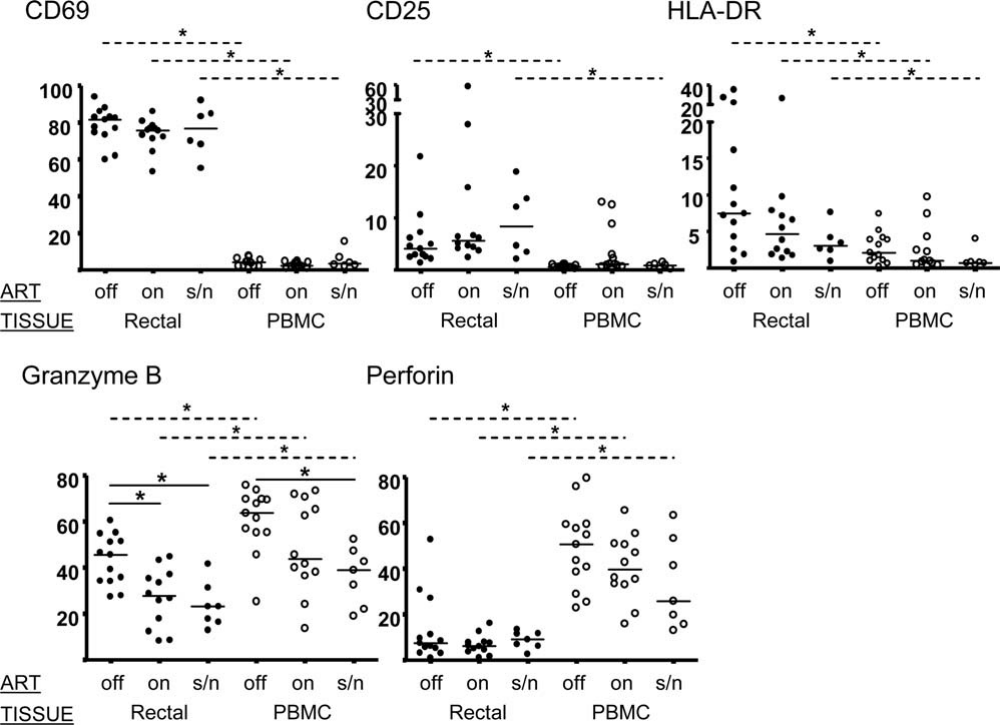
Phenotypic features of CD8+ T-cells from PBMC and rectal mucosa. Unstimulated cells were stained for surface markers and intracellular antigens and analyzed by flow cytometry. The percentage of CD8+ T-cells positive for each marker is given on the Y-axis. Median values are indicated by a horizontal line. Statistically significant differences between groups (*p*<0.05) are denoted by an asterisk with solid line (Kruskal-Wallis) or broken line (Paired T-test). Rectal mucosa (filled circles); PBMC (open circles); ‘off’, not on ART; ‘on’, on ART; s/n, seronegative.

Typical lymphocyte activation markers were generally expressed at higher levels by CD8+ T-cells in rectum than in PBMC (Fig. 8). In agreement with earlier studies [31], the early activation marker CD69 was highly expressed on rectal CD8+ T-cells as compared to PBMC. This high level of expression, approximately 75%, was unaffected by ART and was equally high in the seronegative group. The expression of CD25 was lower in magnitude but similar in expression pattern to that of CD69; this marker was more highly expressed in rectal CD8+ T-cells generally than in PBMC, irrespective of ART or serological status. The activation marker HLA-DR was more strongly expressed in rectal mucosa than PBMC of all three groups, and showed a trend towards higher expression in rectal mucosa of seropositive patients not on ART as compared to other groups.

As previously reported, perforin expression was extremely low in rectal CD8+ T-cells as compared to PBMC [32]; however, three untreated HIV positive patients in this study expressed perforin in >20% of rectal CD8+ T-cells. Granzyme B expression in rectal mucosa CD8+ T-cells of seropositive individuals not on therapy was significantly elevated compared to both subjects on ART and seronegative subjects. This pattern was also evident in PBMC, though in this compartment the effect of ART was not statistically significant.

## Discussion

In this study we introduce the use of a statistical approach to establish when an antigen-specific response is significantly different from the response elicited by costimulatory antibodies alone (see Materials & Methods). While this issue may seem mundane, it is in fact critically important when small populations of responding cells are distributed amongst 31 Boolean combinations, a process that can result in categories containing relatively few events (i.e., individual cells), as is frequently the case for samples derived from tissue biopsies [33]. Further, baseline cytokine production in rectal mucosa is generally higher than in PBMC [26], [34], [35]; this higher background is another reason to apply greater rigor in determining the significance of antigen-specific responses. The method we present here takes into account the absolute numbers of responding cells in a given sample, as well as the total number of cells analyzed. This permits a statistical evaluation on a category-by-category basis of whether an antigen-specific response is significantly different from background. Accordingly, this approach may prove useful for future studies of mucosal immune responses, including for vaccine trials.

Virions in circulation are thought to originate from tissue sites of active virus replication. As the intestinal tract is a major lymphoid organ that supports high levels of virus replication during acute infection, it may also serve as a significant source of viremia during chronic infection. In a previous study of a more limited patient group, we observed that plasma viremia was inversely, though not significantly, associated with IFNγ production by Gag-specific rectal CD8+ T-cells, while blood CD4 count showed a significant positive correlation with Gag-specific rectal responses [26]. Here, by expanding our focus to include additional patients as well as five individual Gag-specific effector responses, we found that three (CD107a, IFNγ, and TNFα) of the five rectal responses were inversely correlated with plasma viral load (p<.05), and two (CD107a and IFNγ) were positively correlated with blood CD4 count (p<.05) (Fig 4) in patients not on ART. When evaluated in terms of polyfunctionality, as defined by the ability to mount 3, 4, or 5 responses simultaneously, significant relationships were also evident between rectal Gag-specific responses and both plasma viral load and blood CD4 count. Thus, our general finding is that greater responsiveness of rectal gag-specific CD8+ T-cells, but not peripheral blood CD8+ T-cells, is associated with lower plasma viral load and elevated blood CD4 count. Determination of whether the relationships between rectal antiviral responses and peripheral viral load are causal will require additional study. The degree to which rectal CD8 cells are responsible for limiting mucosal virus production remains unknown and awaits intensive analysis of tissue viral loads. It is also possible that mucosal CD8 responses are highest and most polyfunctional in individuals with low viremia due to a comparatively intact immune system and the availability of abundant CD4 help.

Others have observed that peripheral Gag-specific CD8+ T-cell responses have clinical relevance as suggested by high-magnitude and complex Gag-specific responses in HIV controllers [12], [36], as well as the ability of Gag-specific CTL to target conserved epitopes in critical Gag functional domains, driving the introduction of CTL escape mutations [18], [37]. In addition, several recent reports have revisited the relationship between peripheral Gag-specific CD8+ T-cell responses and clinical parameters in clade B and C-infected cohorts from North and South America as well as Africa [10]–[16]. Considering these findings and our own, there is emerging support for an association between Gag-specific CD8+ T-cell response magnitude and/or breadth, and clinical parameters such as viral load and blood CD4 count. The significant correlations we identify in the current study, using a relatively small cohort, suggest that HIV-specific CD8+ T-cell activity in intestinal mucosal tissue may be more closely linked to clinical status than CTL activity in peripheral blood alone.

Our finding of enhanced production of MIP-1β and CD107a on a per-cell basis as functionality increases is the first observation of this kind for mucosal CD8+ T-cells yet is consistent with other studies reporting increased IFN-γ production by polyfunctional T-cells in peripheral blood [23]–[25]. Thus, our data lend additional support to the idea that polyfunctional CD8+ T-cells not only engage a greater number of effector functions but also implement them to a higher magnitude. An example of the potential relevance of this is that in the case of HIV infection, MIP-1β production can play a dual role in host defense by blocking HIV infection of prospective target cells and by recruiting CCR5-bearing memory CD8+ T-cells to sites of infection. This dual role may be of particular importance in the mucosal lamina propria, where CD4+ T-cells are rapidly infected during acute infection. It is also noteworthy that MIP-1β and CD107a were the two most highly conserved responses in both rectal mucosa and peripheral blood (Figs 3 & 5A), suggesting that these effector functions play fundamentally important roles in the antiviral repertoire.

It has been known for some time that the magnitude of HIV-specific CD8+ T-cell responses in peripheral blood declines rapidly when individuals begin ART [38]. In contrast, very little information is available on the effects of ART on peripheral CTL response quality, or on mucosal CTL functionality overall. Our data reveal that ART is associated with a reduced total response magnitude in both blood and mucosa, as expected based on previous studies, but the complexity of the response is affected differentially in the two tissues (Fig 5A & B). In blood, the response complexity was not different relative to ART status, an observation which is consistent with a recent report [39]. In contrast, the drastic reduction in rectal response complexity associated with ART is a unique observation and suggests that ART exerts more complex effects on mucosal CTL functionality. Longitudinal studies of individuals on ART will be more revealing and are underway in our laboratory; findings from these studies will be presented elsewhere.

Immune reconstitution in the context of ART, particularly as defined by CD4 repopulation in the intestinal tract and peripheral blood, is of intense current interest. Previously we reported a significant positive correlation between CD4 percentages in these two compartments [26]; here, we discriminate between subject groups and find that only the group on ART shows a significant correlation between values in the two tissues. It is possible that this relationship develops out of ART-induced reconstitution of CD4+ T-cells in peripheral blood and/or the intestine, though the cross-sectional design of this study did not allow direct evaluation of CD4 reconstitution. Our analysis of eight seronegative individuals showed no correlation between peripheral and rectal mucosal CD4+ T-cell percentages, with values for both tissues being much more tightly clustered than in infected subjects.

In summary, we have shown here that rectal HIVgag-specific CD8+ T-cells from chronically infected subjects not on ART are able to mount robust and diverse effector responses which correlate positively with blood CD4 count and inversely with plasma viral load. These associations were not evident for peripheral blood CD8+ T-cells, suggesting a unique status for mucosal HIV-specific CD8+ T-cells which remains to be fully defined. In the context of ART, a predictable drop in the total responding CD8+ T-cells in both tissues was accompanied by a marked loss of response complexity in rectal mucosa. The provision of ART was also linked to the observation that peripheral and rectal CD4+ T-cell percentages are tightly positively correlated.

Although a role for mucosal T-cell responses in limiting viral dissemination during early/acute infection has been postulated, the contribution of mucosal effector cells to the immune control of viral replication during chronic infection has not been addressed. The results presented here suggest that, when individuals throughout a broad range of viral loads and blood CD4 counts are evaluated, there are significant relationships between antiviral mucosal effector responses (both individually and in combination), and both VL and CD4. However, a cause/effect relationship between mucosal CD8+ T-cells and clinical status cannot be definitively established due to the cross-sectional nature of this study; indeed, it is possible that polyfunctional responses are a consequence of an intact immune system in individuals with low viral replication, rather than the cause of low virus replication. Additional studies will be required to address the relationship between mucosal T-cell responses and tissue viral loads.

## Materials and Methods

### Study participants, clinical procedures, and tissue processing

Subjects were chronically HIV-infected patients and seronegative volunteers and at the Center for AIDS Research, Education and Services (CARES), Sacramento, CA. Patients taking and not taking antiretroviral drugs were enrolled in the study. This patient cohort has been described previously [26]; however, the tissue samples and the analytical results presented for this study are entirely new and have not been previously reported. Written informed consent for phlebotomy and rectal biopsy was obtained through study protocols approved by the Institutional Review Board, School of Medicine, University of California, Davis. Blood was collected by sterile venipuncture using EDTA as an anticoagulant. Twenty-four rectal biopsies were taken endoscopically approximately 10–15 cm from the anal verge as previously described [26]. Tissue pieces were collected and immediately placed in complete media (described below). Rectal biopsies and blood were collected at the same visit to the clinic and processed the day of collection.

PBMC were isolated from blood using Ficoll-Paque™ (Pfizer-Pharmacia, New York, NY). Isolation of mononuclear cells from rectal biopsies was done using an optimized collagenase digestion procedure as previously described [26], [40]. Following isolation, PBMC and rectal mononuclear cells (RMC) were incubated overnight in complete medium [RPMI-1640 supplemented with fetal calf serum (15%), penicillin (100 U/ml), streptomycin (100 µg/ml), and glutamine (2 mM)]. Rectal cells were further treated with Piperacillin-Tazobactam (0.5 mg/ml) (Zosyn®, Wyeth Pharmaceuticals, Philadelphia, PA) to inhibit overgrowth of intestinal bacteria that may be present in rectal cell preparations.

### Flow cytometry, blood CD4 and plasma viral loads

Flow cytometry data were acquired on an LSRII (BD Immunocytometry Systems, San Jose, CA) equipped with 405, 488, and 643 nm lasers and utilizing FACSDIVA software (BDIS). Analysis of cytometry data was done with FlowJo software (Tree Star, Ashland, OR); for experiments measuring five functional responses, individual cytokine gates were evaluated alone and also processed through Boolean combinations to partition responding cells into one of 31 possible specific response categories. Cytometry data were biexponentially transformed in order to include all events. SPICE software (Mario Roederer, Vaccine Research Center, NIAID/NIH, Bethesda, MD) was used to graph response data.

Blood CD4 count was determined by the clinical laboratory at University of California, Davis, Medical Center. Plasma viral load was measured by the California Department of Health Services, Viral and Rickettsial Disease Laboratory using the Roche COBAS HIV-1 Monitor v1.5 test. This method has a sensitivity cutoff of 50 HIV-1 RNA copies per ml plasma.

### Antibodies and viral peptides

Fluorochrome-labeled monoclonal antibodies to CD3 (clone UCHT1), CD107a (clone H4A3), IFNγ (clone B27), TNF-α (clone MAb11), IL-2 (clone 6344.111), MIP-1β (clone D21-1351), Granzyme B (clone GB11), and CD25 (clone M-A251) were purchased from Pharmingen (San Diego, CA), as were unlabeled CD28 (clone L293) and CD49d (clone L25), and 7-Amino-Actinomycin D (7-AAD). Fluorochrome-labeled aniti-CD8 (clone 3B5), CD4 (clone S3.5), HLA-DR (clone TU36), and CD69 (clone CH/4) were purchased from Invitrogen. Anti-CD4 (clone SFCI12T4D11) was obtained from Beckman Coulter (Fullerton, CA) and anti-Human Perforin (clone PF-164) was obtained from MABTECH. The HIVgag peptide pool (p55, HXB2 sequence) consisted of 15-mers with an 11 amino acid overlap (BD Biosciences, San Jose, CA).

### Intracellular cytokine staining (ICS), T-cell phenotyping, and Flow cytometry

ICS assays and phenotypic staining were carried out on freshly isolated PBMC and RMC that were rested overnight at 37°C, 5% CO_2_ prior to analysis. For ICS, cells were stimulated with pooled HIVgag peptides (3.5 µg/ml) plus costimulatory antibodies (CD28 and CD49d). Negative and positive controls were media containing peptide vehicle (DMSO) plus costimulatory antibodies, and staphylococcus enterotoxin B (SEB, 5 µg/ml), respectively. The expression of CD107a, MIP-1β, IL-2, TNF-α and IFN-γ were measured using a modification of a previously described method [26], [27]. Briefly, 1×10^6^ cells in 200 µl complete medium were treated with anti-CD28 (2.5 µg/ml), anti-CD49d (5 µg/ml), anti-CD107a, monensin (1 µM GolgiStop™, BD Biosciences), brefeldin A (5 µg/ml, Sigma), and HIVgag peptide pool or DMSO. Following a 5 hour incubation, cells were incubated for 5 minutes in PBS/2% FCS/0.5 mM/EDTA, stained for surface markers and 7-AAD (Pharmingen) in PBS/2% FCS for 20 min at 4°C, fixed in 4% formaldehyde, then permeabilized using FACS Perm 2 (BD Biosciences). Cells were then washed in PBS/2% FCS, stained for intracellular markers and CD3, washed again, then stored at 4° C in PBS/1% formaldehyde until analysis (within 24 hours). Non-fluorescent Actinomycin D (2 µg/ml) was included in reagents after the 7-AAD staining step to saturate sites that could bind residual 7-AAD [41], [42].

Phenotypic staining of unstimulated peripheral and rectal CD8+ T-cells for surface and intracellular antigens was carried out using Caltag Fix and Perm (Invitrogen) according to the manufacturer's protocol. Stained cells were stored at 4° C in PBS/1% formaldehyde until analysis (within 24 hours). Flow cytometry gating was as shown in Fig. 1, except that the endpoint gates were phenotypic markers rather than functional responses.

### Determination of the statistical significance of net antigen-specific responses

Net antigen-specific responses were evaluated using a statistical test [43] to determine whether the percent of CD8+ T-cells responding to Gag plus costimulation differed significantly from the percent of CD8+ T-cells responding to costimulation alone. This step was considered critical to the analysis given the observation that mucosal lymphocytes are partially activated *in vivo* and frequently exhibit elevated cytokine production relative to PBMC [26], [34], [44], [45]. The statistical test was based on the assumption of a Poisson distribution for both conditions, and calculations utilized the number of gated fluorescence events collected, rather than percentages. This was considered advantageous given the fact that mucosal cell isolation protocols generally yield a lower number of viable lymphocytes than PBMC isolation protocols [40]. Within this framework, a choice of tests was made that generally controls the significance level of the test to the nominal level [43]. The following formula shows how the statistical test was structured to accommodate flow cytometry response data:
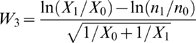
The value *X_1_* is the number of cells (i.e., positive fluorescence events) responding to peptide plus costimulation within a total CD8+ T-cell population designated as *n_1_*. Similarly, *X_0_* is the number of events associated with costimulation alone within a total CD8+ T-cell population designated as *n_0_*. The test was carried out as follows: (1) If one or both of *X_0_* and *X_1_* was equal to zero, then the value was set to 0.5; (2) the test statistic *W_3_* was calculated; (3) the test statistic *W_3_* was referred to a standard normal distribution and the *p*-value was calculated; (4) If the *p*-value was less than the significance level (0.05), this indicated that the background cytokine response and the Gag-stimulated cytokine response were statistically different. In this case, the background response was subtracted from the Gag-specific response to yield a statistically meaningful net response. If, on the other hand, the *p*-value was equal to or greater than 0.05, this indicated that the background cytokine response and the Gag-stimulated response were not statistically different. In this case, the net Gag-specific response was set to zero.

### Statistical comparisons between tissues and between subjects

For comparisons of T-cell function between tissues within the same patients, paired T-tests (two-tailed) were used. Mann Whitney (two-tailed) tests were used to compare T-cell function data between subjects (i.e., on vs. off ART) within a given tissue type. Expression of phenotypic markers was compared between tissues within the same patients using a paired T-test, and between patient groups using a Kruskal-Wallis test followed by Dunn's Multiple Comparison Test. Correlations between CD8+ T-cell response magnitudes and plasma viral load, CD4+ T-cell count (blood) and CD4+ T-cell frequency (rectal MNC) were determined using Spearman Correlation (GraphPad Prism, v5, Graph Pad Software, San Diego, CA). Linear regression analysis was used to display a best fit line to the data.

### Linear mixed model analysis of cytokine responses

T-cell responses to antigenic stimulation were evaluated by Boolean partitioning of responses into 31 distinct responding populations [22], [28]. For the purposes of linear mixed model analysis, we defined polyfunctional cells as those capable of 3, 4, or 5 responses, as distinct from mono- or dual-function cells. Percentage values were square root transformed for variance stabilization. The transformed data were analyzed using a linear mixed effect model with ART status, tissue type, and polyfunctionality as fixed effects (including all interactions), with a random effect for subject, and with unstructured correlation between the tissue and polyfunction measures within each subject. The model was estimated using restricted maximum likelihood, and degrees of freedom were calculated using the Kenward and Roger adjustment [46]. Post-hoc comparisons were performed using the Tukey-Kramer adjustment for multiple comparisons. The level of significance was set at 0.05. Model fit was assessed using graphical analysis of residuals and the Shapiro-Wilks test for normality. Analysis was implemented using the MIXED procedure in SAS for Windows, Version 9.1.
